# 1668. The SAFER Lines Project: A Mobile-App Strategy for Prevention of Outpatient Central Line Associated Bloodstream Infection (CLABSI)

**DOI:** 10.1093/ofid/ofac492.134

**Published:** 2022-12-15

**Authors:** Shruti K Gohil, Bardia Bahadori, Shereen Nourollahi, Thomas T Tjoa, Hiroki Saito, Jessica Bethlahmy, Mohamad Alsharif, Justin Chang, Edward L Nelson, Richard A Van Etten, Linda Armendariz, Vincent Torres, Sandra Masson, Syma Rasheed, Marlene Esteves, Raheeb Saavedra, Raveena D Singh, Susan S Huang

**Affiliations:** University of California, Irvine, Irvine, CA; University of California, Irvine, Irvine, CA; University of California, Irvine, Irvine, CA; University of California, Irvine School of Medicine, Division of Infectious Diseases, Irvine, California; St. Marianna University School of Medicine, Yokohama City Seibu Hospital, Yokohama, Kanagawa, Japan; University of California, Irvine, Irvine, CA; University of California, Irvine, Irvine, CA; University of California, Irvine, Irvine, CA; University of California, Irvine, Irvine, CA; University of California, Irvine, Irvine, CA; University of California, Irvine, Irvine, CA; University of California, Irvine, Irvine, CA; University of California, Irvine, Irvine, CA; University of California, Irvine, Irvine, CA; University of California, Irvine, Irvine, CA; University of California, Irvine School of Medicine, Division of Infectious Diseases, Irvine, California; University of California, Irvine School of Medicine, Division of Infectious Diseases, Irvine, California; University of California, Irvine School of Medicine, Irvine, CA

## Abstract

**Background:**

Outpatient peripherally inserted central catheters (PICC) use has grown without standardized protocols for their management. We assessed the impact of a mobile app strategy for outpatient CLABSI prevention using photo-monitoring, assessment, and response to lines with local inflammation/infection in a cohort of cancer clinic patients.

**Methods:**

This prospective cohort study evaluated adults with PICCs at an academic cancer clinic at baseline (7/2015–12/2016) and after implementing the SAFER (Standardizing Assessment For Effective Response) Lines program (intervention 5/2017–11/2018). This included a mobile app enabling (1) clinic assessment of localized inflammation or infection defined as Central Line Insertion Site Assessment (CLISA) score 2 or 3, respectively (Table 1), (2) photo-documentation, and (3) score-based automated physician alerts for remote response. We assessed demographics, malignancy type, and line characteristics. Generalized linear mixed effects model assessed program impact on frequency of CLISA 2 or 3 lines, clustered by patient. Cox proportional hazards and Kaplan Meier models assessed days to line removal after CLISA 2 or 3 were identified.

**Results:**

Among 4,894 assessments of 528 PICCs in 380 outpatients, there were 272 lines (199 patients) at baseline and 256 lines (181 patients) after SAFER program implementation. Mean age, gender, PICC dwell time, and history of prior PICC were similar at baseline and intervention. The proportion of inflamed (CLISA 2) and infected (CLISA 3) lines decreased 40% (from 26% to 16%, and 19% to 11%, respectively) during intervention compared to baseline. Lines with peeling dressings decreased 80% (from 46% to 9%). Mean days to removal of inflamed lines decreased 59% (from 19 to 8 days); removal of infected lines decreased 85% (from 11 to < 2 days). Intervention was associated with 46% lower risk of local inflammation/infection (OR 0.46, CI=0.26–0.83, p< 0.01, Table 2) and faster line removal when such lines were identified (HR 0.18, CI=0.12–0.27, p< 0.01).

**Conclusion:**

The SAFER Lines mobile app and program decreased the frequency of locally inflamed or infected PICC insertion sites and increased the speed of removal when local inflammation/infection was found in cancer clinic patients.

**Disclosures:**

**Raheeb Saavedra, AS**, Medline: Conducted studies in which hospitals and nursing homes received contributed antiseptic and/or environmental cleaning products|Stryker: Conducted clinical studies in which hospitals and nursing homes received contributed antiseptic products|Xttrium Laboratories: Conducted clinical studies in which hospitals and nursing homes received contributed antiseptic products **Raveena D. Singh, MA**, Medline: Conducted studies in which hospitals and nursing homes received contributed antiseptic and/or environmental cleaning products|Stryker: Conducted clinical studies in which hospitals and nursing homes received contributed antiseptic products|Xttrium Laboratories: Conducted clinical studies in which hospitals and nursing homes received contributed antiseptic products **Susan S. Huang, MD, MPH**, Medline: Conducted studies in which hospitals and nursing homes received contributed antiseptic and/or environmental cleaning products|Molnlyke: Conducted clinical studies in which hospitals received contributed antiseptic product|Stryker: Conducted clinical studies in which hospitals and nursing homes received contributed antiseptic products|Xttrium Laboratories: Conducted clinical studies in which hospitals and nursing homes received contributed antiseptic product.

